# The Use of ZnO Quantum Dots to Improve the Electrical Properties of Silicon Solar Cells

**DOI:** 10.3390/ma18040861

**Published:** 2025-02-16

**Authors:** Magdalena Monika Szindler, Marek Szindler, Krzysztof Lukaszkowicz, Krzysztof Matus, Mateusz Fijalkowski, Tomasz Węgrzyn, Bożena Szczucka-Lasota, Jakub Polis

**Affiliations:** 1 Department of Engineering Materials and Biomaterials, Faculty of Mechanical Engineering, Silesian University of Technology, Konarskiego 18a, 44-100 Gliwice, Poland; magdalena.szindler@polsl.pl (M.M.S.); jakupol064@student.polsl.pl (J.P.); 2 Scientific and Didactic Laboratory of Nanotechnology and Material Technologies, Faculty of Mechanical Engineering, Silesian University of Technology, Towarowa 7, 44-100 Gliwice, Poland; marek.szindler@polsl.pl; 3 Materials Research Laboratory, Faculty of Mechanical Engineering, Silesian University of Technology, Konarskiego 18a, 44-100 Gliwice, Poland; krzysztof.matus@polsl.pl; 4 Institute for Nanomaterials, Advanced Technologies and Innovation, Technical University of Liberec, Studentská 1402/2, 461 17 Liberec, Czech Republic; mateusz.fijalkowski@tul.cz; 5 Faculty of Transport and Aviation Engineering, Silesian University of Technology, Krasinskiego 8, 40-019 Katowice, Poland; tomasz.wegrzyn@polsl.pl (T.W.); bozena.szczucka-lasota@polsl.pl (B.S.-L.)

**Keywords:** quantum dots, ZnO, silicon solar cells, HRTEM, LBIC

## Abstract

Silicon-based solar cells dominate the photovoltaic market, with commercial monocrystalline silicon cells reaching efficiencies as high as 27.3% by May 2024. An alternative to monocrystalline silicon solar cells is polycrystalline solar cells. Despite their lower efficiency (record: 23.81%), their manufacturing process is simpler and cheaper, and their energy conversion efficiency is less sensitive to temperature changes. However, limitations persist in optical and electrical losses, particularly underutilizing ultraviolet (UV) radiation due to silicon’s bandgap. To address these issues, the application of down-converting materials like zinc oxide (ZnO) quantum dots (QDs) has gained attention. ZnO QDs absorb high-energy UV light and re-emit it in the visible spectrum, optimizing the portion of solar energy usable by silicon cells. This study explores the synthesis of ZnO QDs using a sol–gel method, followed by their application on polycrystalline silicon solar cells. Experimental results indicated an increase in short-circuit current and overall efficiency, with the efficiency rising from 18.67% to a maximum of 19.05% when ZnO QDs were deposited from a 5 mg/mL solution. These findings suggest that ZnO QDs could significantly enhance solar energy conversion efficiency by utilizing portions of the solar spectrum that would otherwise be wasted.

## 1. Introduction

Currently, several technologies are used in photovoltaic (PV) energy generation, each with its unique characteristics and advantages. The most common and widely adopted technology is silicon-based PV, which includes both monocrystalline and polycrystalline silicon cells. These cells are highly efficient and well established in the market. In addition to silicon, there are other notable technologies, such as thin-film solar cells. These cells are made by depositing one or more layers of photovoltaic material on a substrate, which makes them lightweight and flexible. Thin-film technologies include cadmium telluride (CdTe), amorphous silicon (a-Si), and copper indium gallium selenide (CIGS). Another emerging technology is dye-sensitized solar cells (DSSCs), which use organic dyes and a semiconductor material to convert light into electricity. This technology offers potential benefits in terms of cost and flexibility, although its efficiency currently lags behind traditional silicon cells. Each of these technologies continues to evolve, with ongoing research aimed at improving efficiency, reducing costs, and enhancing the scalability of solar energy production [1,2,3,4,5,6,7,8].

In addition to traditional photovoltaic technologies, recent advancements in engineered architectures containing nanostructured semiconductor materials are promising for developing highly efficient, energy-conversion photovoltaic systems. These novel designs incorporate various nanomaterials, such as quantum dots, nanowires, and nanorods, to enhance the absorption and conversion of light into electricity. These architectures aim to improve power conversion efficiency while maintaining cost-effectiveness and scalability. The incorporation of nanostructured materials enables better light trapping, charge separation, and reduced recombination, leading to higher efficiency than conventional systems. These promising systems, while still in the research and development phase, could be key to achieving the next generation of high-performance, energy-efficient solar cells [1,2,3,4,5,6,7,8,9,10].

Presently, the solar energy market is predominantly driven by silicon-based solar cells. Silicon, the second most abundant chemical element on Earth, is well understood due to its significant role in electronics. The rapid growth of the photovoltaic industry, which primarily uses crystalline silicon cells, has led to the development of advanced manufacturing techniques that optimize production while minimizing silicon consumption [1,2,3]. Monocrystalline silicon solar cells in mass production typically achieve an efficiency of 21.4%, demonstrating reliable performance over extended operational periods. As of May 2024, the efficiency of commercial monocrystalline silicon solar cells reached a new milestone of 27.3%. An alternative to monocrystalline silicon solar cells is polycrystalline solar cells due to their lower production costs and simpler manufacturing process. While they generally have lower efficiency, recent advancements have improved their performance. The current record for polycrystalline silicon cells stands at 23.81%, demonstrating significant progress. Additionally, they are less sensitive to temperature variations, making them suitable for diverse environments. Their cost-effectiveness and evolving efficiency make them a viable option for solar energy applications. Despite these advancements, further research is needed to develop emerging solar cell technologies. A critical driver for accelerating progress in this field involves broadening the range of solar cell applications while reducing manufacturing costs. Silicon solar cells, while widely used, face several inherent challenges related to optical and electrical losses. These losses occur due to multiple factors, including the reflection of incoming light, the recombination of charge carriers, and thermalization, where excess energy from high-energy photons is dissipated as heat rather than converted into usable electricity [4,5,6,7,8].

One fundamental limitation is that silicon can only effectively utilize a specific portion of the solar spectrum. This limitation is governed by the bandgap of silicon, which restricts the absorption of photons below a certain energy threshold, leaving a significant fraction of the solar spectrum unexploited for energy conversion. To mitigate these losses, various strategies have been developed, one of which involves the application of a phosphor layer to the solar cell’s surface [11,12,13]. This layer plays a crucial role in enhancing the quantum efficiency of the solar cell by converting higher-energy photons, typically in the ultraviolet (UV) range, into lower-energy photons that silicon can more effectively absorb. This process, known as down-conversion, allows the cell to utilize parts of the solar radiation that are otherwise ineffective in generating electricity directly. In addition, UV radiation causes a decrease in solar cell efficiency during its use (combined with progressive degradation of the system). A phosphor is a material that can absorb high-energy radiation, typically within the shorter wavelength range, and re-emit it as lower-energy radiation in the longer wavelength range. This property is highly beneficial in photovoltaic applications, where the goal is to convert as much solar radiation as possible into usable energy [11,12,13,14,15,16].

A classic example of such a phosphor is the tris-(8-hydroxyquinoline) aluminum complex, called Alq-3. This compound absorbs ultraviolet (UV) light at a wavelength of around 390 nm and re-emits it as visible light at 519 nm, making it useful for applications that involve converting high-energy UV radiation into light that can be absorbed by conventional solar cells. Silicon, the most commonly used material in solar cells, efficiently absorbs light with wavelengths shorter than 400 nm. However, due to high surface recombination, much of the energy from the UV photons is lost rather than contributing to electricity generation. This limitation makes the use of luminophores beneficial, as they can convert high-energy UV photons into longer wavelengths that silicon cells can utilize more effectively. This limitation means that UV radiation is not used effectively [17,18,19,20]. By incorporating a phosphor such as Alq-3, photons in the UV range (below 400 nm) can be absorbed and subsequently re-emitted as photons with a longer wavelength, generally above 500 nm. These re-emitted photons fall within the absorption range of silicon, allowing the solar cell to utilize additional radiation that would otherwise be wasted.

Another promising alternative to organic phosphors like Alq-3 is the use of quantum dots, particularly zinc oxide quantum dots (ZnO QDs). ZnO QDs exhibit strong absorption in the UV range, particularly below 350 nm, and emit light in the visible spectrum between 520 and 560 nm, depending on the size of the quantum dot core and the stabilizing layer. These characteristics make ZnO QDs a potential candidate for improving the efficiency of silicon-based solar cells [19,20,21,22,23]. The emission wavelength of ZnO quantum dots can be finely tuned by controlling their size and surface chemistry. This tunability allows for greater flexibility in optimizing their luminescent properties for specific solar cell applications. Compared to organic compounds like Alq-3, ZnO quantum dots offer better thermal and chemical stability. Furthermore, their inorganic nature reduces the likelihood of degradation over time, making them a more durable option for long-term photovoltaic applications. The stabilizing layers that surround the ZnO QDs also play a crucial role in determining their optical properties. Choosing the appropriate stabilizers makes it possible to fine-tune the emission characteristics to match the absorption profile of the underlying silicon solar cell. Integrating ZnO quantum dots into photovoltaic devices can increase overall energy conversion efficiency by utilizing portions of the solar spectrum that would otherwise be lost. Additionally, their compatibility with various deposition techniques, such as solution processing or thin-film formation, makes them easier to integrate into existing manufacturing processes [22,23,24,25,26].

Quantum dots (QDs) that enhance power conversion efficiency (PCE) cover a range of types, often found in chemical and biological applications, including Si-QDs, CQDs, CdS, CdSe, CdTe, CuInS_2_, CuS, PbS, PbSe, CdSe/ZnS, ZnS, InP, InAs, Ag_2_S, Bi_2_S_3_, Sb_2_S_3_, and CuInS_2_/ZnS. Deposition techniques vary by the substrate and can include methods like spin coating, drop-casting, inkjet printing, plasma-enhanced chemical vapor deposition (PECVD), and spin-on-glass, each selected based on compatibility with the host material [27,28,29,30]. For example, the I-V characterization of silicon crystalline cells before and after the application of phenylacetylene-capped (PA) Si-QDs showed average increases in the short-circuit current (I_sc_) of 0.75% and 1.06%, respectively. This improvement demonstrated the effectiveness of PA Si-QDs in enhancing I_sc_, highlighting their potential for boosting the overall efficiency of silicon-based solar cells [31].

This paper proposes further research on the use of ZnO quantum dots (ZnO QDs) on the surface of polycrystalline silicon solar cells as a promising solution for improving their performance. One of the primary reasons for selecting ZnO QDs is the simplicity and cost-effectiveness of their synthesis via the sol–gel method. This technique is advantageous because it does not require complex equipment or high financial investment. Moreover, the sol–gel process operates at relatively low temperatures, making it an energy-efficient approach for producing nanomaterials. Due to these benefits, the sol–gel method remains a widely adopted and contemporary approach in material engineering, particularly in the development of surface modifications and nanotechnology applications. ZnO QDs are particularly suitable for photovoltaic applications due to their optical properties, chemical stability, and ease of production. Their ability to absorb UV light and emit visible light, along with their resistance to degradation, makes them an ideal candidate for enhancing the efficiency of solar cells [23,24,25,26]. Thus, the combination of efficient synthesis methods and favorable material properties justifies this study’s selection of ZnO QDs.

In summary, this paper highlights the potential of ZnO quantum dots (ZnO QDs) as a promising solution to enhance the performance of polycrystalline silicon solar cells. Despite silicon’s efficient UV absorption, its limitations in energy conversion due to surface recombination and poor utilization of UV light are addressed by ZnO QDs. These quantum dots efficiently absorb UV light and re-emit it as visible light, improving energy conversion. Additionally, ZnO QDs offer advantages such as chemical stability, ease of synthesis through cost-effective methods like sol–gel, and compatibility with existing manufacturing techniques, making them a viable candidate for advancing solar cell technology.

## 2. Materials and Methods

Zinc oxide (ZnO) nanopowder was synthesized using zinc acetate dihydrate (purity: ≥99%; Merck, Darmstadt, Germany) as a precursor. Initially, a solution of zinc acetate dihydrate in isopropanol (Merck, ≥99.5%) was prepared and stirred using a magnetic stirrer. Simultaneously, a solution of deionized and distilled water (Merck) was also prepared. These two solutions were combined and stirred magnetically at 50 °C for 24 h. A calcination process was then conducted to remove residual water by heating the compound below its melting point. Stable nanoparticle suspensions were then formulated in dimethyl sulfoxide (DMSO) (purity: ≥99%; Merck, Darmstadt, Germany) at concentrations of 5 mg/mL and 10 mg/mL.

**Figure 1:**
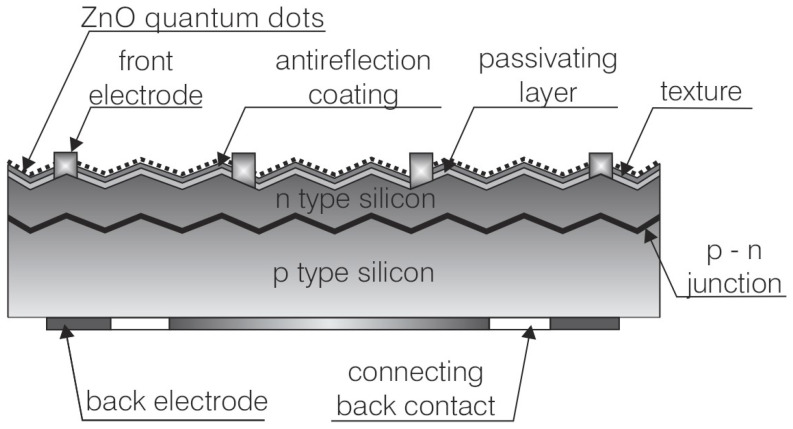
Schematic diagram of a solar cell with fabricated quantum dots.

The synthesized nanopowder underwent structural characterization using transmission electron microscopy (TEM). The analysis was conducted with a high-resolution transmission electron microscope S/TEM Titan 80-300 (Field Electron and Ion Company, Hillsboro, OR, USA) equipped with a super-twin lens, operating at an accelerating voltage of 300 kV. Observations were performed in conventional TEM mode and scanning transmission electron microscopy (STEM) mode. The Titan 80-300 microscope is outfitted with the following three coaxial detectors designed explicitly for STEM: bright field (BF), annular dark field (ADF), and high-angle annular dark field (HAADF). For this analysis, the nanopowder was deposited onto specialized copper grids designed for electron microscopy. Then, zinc oxide nanopowders were deposited on glass substrates. Scanning electron microscopy (SEM) images were acquired using a Zeiss Supra 35 microscope (Carl Zeiss NTS GmbH, Oberkochen, Germany). Additionally, qualitative chemical composition analysis was carried out using an energy dispersive spectroscopy (EDS) system (EDAX Inc., Mahwah, NJ, USA). The absorbance of the zinc oxide nanoparticles was measured using a Thermo Scientific Evolution 220 spectrophotometer (Thermo Fisher Scientific, Waltham, MA, USA) equipped with a xenon lamp over a wavelength range of 190 nm to 1100 nm. The photoluminescence spectra (PL) were measured with an F-4600 spectrofluorometer (Hitachi, Chiyoda, Japan) with an excitation wavelength of 325 nm at room temperature. ZnO quantum dots were deposited onto the surfaces of 5 cm × 5 cm polycrystalline silicon solar cells using the spray coating method (Figure 1).

Two different concentrations of ZnO quantum dots in dimethyl sulfoxide (DMSO) were used—5 mg/mL and 10 mg/mL. The deposition process was carried out at room temperature with the spray nozzle positioned at a distance of approximately 15 cm from the surface of the solar cells. The nozzle was moved at a speed of 2 cm/s to ensure uniform and consistent coverage across the entire surface of the solar cells, each of which had a single silver busbar. The spray coating process was carefully controlled to achieve a uniform distribution of the quantum dots, aiming to enhance light absorption and improve the overall performance of the solar cells. The current–voltage (I-V) characteristics of polycrystalline solar cells were measured using an SS150AAA solar simulator (PV Test Solutions Tadeusz Zdanowicz, Wrocław, Poland). The measurements were conducted under standard test conditions (irradiance P_in_ = 1000 W/m^2^, AM1.5G spectrum, T = 25 °C). Key electrical parameters of the solar cells were determined using the I-V Curve Tracer software (version V 2.18.0.5) The short-circuit current distribution across the cell was analyzed using the Correscan equipment in Light Beam Induced Current (LBIC) mode (SunLab BV, Petten, The Netherlands).

## 3. Results and Discussion

**Figure 2:**
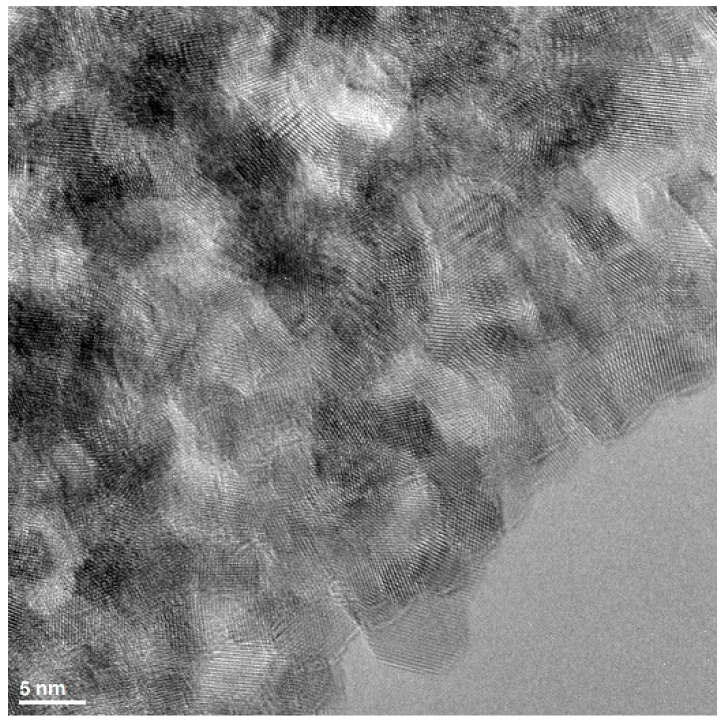
HRTEM image of the synthesized zinc oxide nanoparticles.

The structural characterization of the synthesized zinc oxide (ZnO) nanopowder was conducted using scanning/transmission electron microscopy (S/TEM) techniques to gain insight into its morphology, crystallinity, and elemental composition. High-resolution TEM (HRTEM) imaging revealed that the ZnO nanoparticles exhibit a polycrystalline nature, with clearly visible lattice fringes indicative of their well-defined crystalline structure. The nanoparticles possess diameters of less than 10 nanometers, which suggests their potential applicability in nanoscale electronic and optical devices (Figure 2).

**Figure 3:**
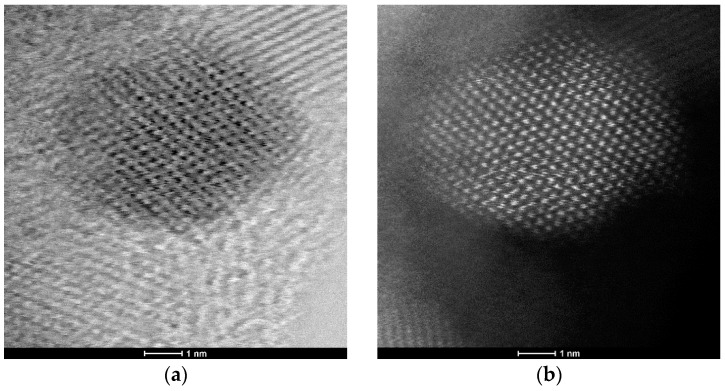
Images of the synthesized zinc oxide nanoparticles: (**a**) STEM-BF; (**b**) STEM-HAADF.

**Figure 4:**
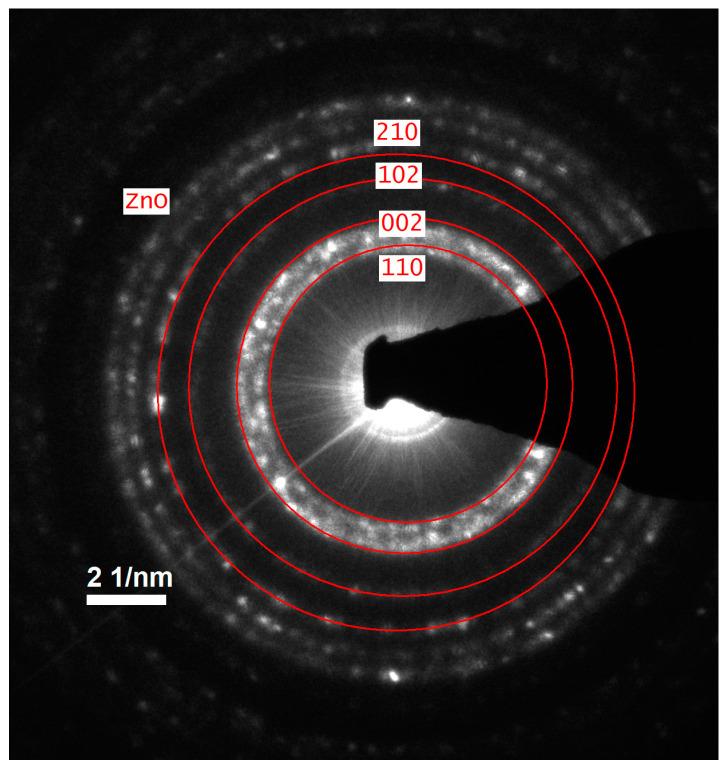
The SAED pattern of hexagonal ZnO nanoparticles.

**Figure 5:**
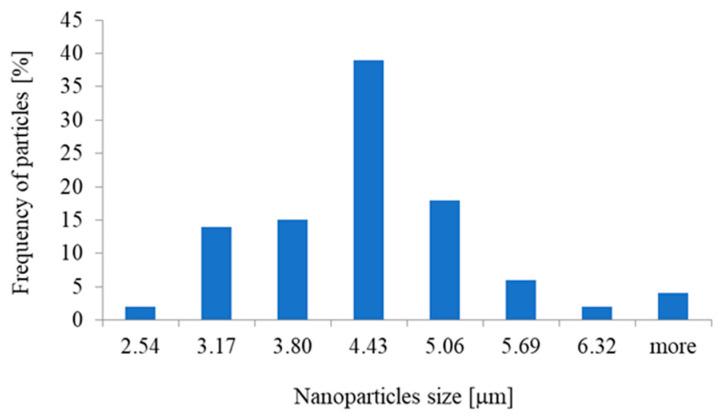
Distribution of zinc oxide nanoparticle sizes.

**Figure 6:**
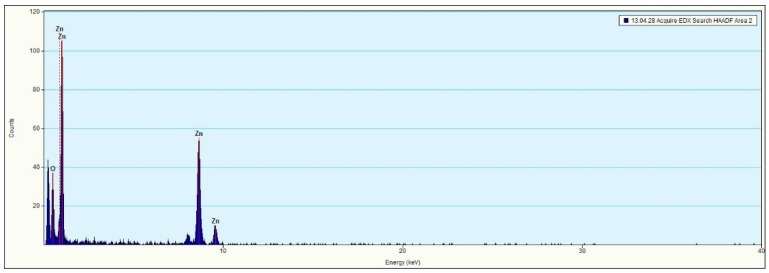
EDS spectrum of zinc oxide nanoparticles.

In addition to HRTEM imaging, the study utilized the scanning transmission electron microscopy (STEM) mode to obtain complementary structural information. The Titan 80-300 microscope, equipped with bright field (BF), annular dark field (ADF), and high-angle annular dark field (HAADF) detectors, was employed to enhance contrast and elucidate the internal structure of the nanoparticles. The BF-STEM mode highlighted variations in density, whereas the ADF and HAADF-STEM images provided Z-contrast, confirming the uniform distribution of ZnO nanoparticles and their crystalline features (Figure 3a,b). The selected area electron diffraction (SAED) pattern further validated the hexagonal wurtzite structure of ZnO, as evidenced by distinct diffraction rings corresponding to the expected crystallographic planes (Figure 4). A statistical analysis of particle size distribution was conducted based on multiple recorded images, and the resulting histogram demonstrated a relatively narrow size dispersion, with most particles measuring below 10 nm (Figure 5). Furthermore, energy-dispersive X-ray spectroscopy (EDS) confirmed the elemental composition of the synthesized ZnO nanoparticles, verifying the presence of zinc and oxygen without significant impurities (Figure 6).

The histogram presented in Figure 5 demonstrates the size distribution of zinc oxide nanoparticles, revealing valuable insights into their dimensional characteristics. The majority of particles are concentrated in the range of 4.43 ± 0.3 μm, indicating that this size range is the most prevalent in the sample. The distribution is notably asymmetric, with a higher frequency of smaller particles and fewer larger ones. This suggests that the synthesis process predominantly generates nanoparticles within a specific size range, but there is also a tail of larger particles. The observed asymmetry could be indicative of agglomeration or secondary growth of particles, leading to a slight presence of larger sizes, which may influence the material’s properties and performance.

The structural characteristics of the synthesized ZnO nanoparticles in this study align well with findings reported in the literature. High-resolution transmission electron microscopy (HRTEM) analyses commonly reveal that ZnO nanoparticles exhibit a polycrystalline nature with a hexagonal wurtzite crystal structure. For instance, a study published in *ACS Omega* reported that synthesized ZnO nanoparticles displayed a hexagonal wurtzite crystal arrangement, as identified through various characterization techniques, including transmission electron microscopy (TEM) [32]. Furthermore, the particle size distribution observed in our study, with diameters of less than 10 nanometers, is consistent with other research findings. In the same *ACS Omega* study, the ZnO nanoparticles exhibited similar nanoscale dimensions, which were confirmed through XRD analysis [33]. These comparisons indicate that our synthesized ZnO nanoparticles share structural characteristics with those reported in the existing literature, supporting the validity and reproducibility of our synthesis and characterization methods.

The chemical sol–gel method employed in the preparation of ZnO QDs offers significant control over the size, morphology, and reproducibility of the nanostructures. Based on the TEM studies and the obtained images, as well as the size distribution histogram, we demonstrate the ability to achieve a consistent and narrow size distribution of the QDs. This approach allows for a precise tuning of the synthesis conditions, ensuring reproducible results in the formation of uniform nanostructures. The high level of control achieved through this method is crucial for the reliable fabrication of ZnO QDs, highlighting its potential for scalable production and diverse applications.

**Figure 7:**
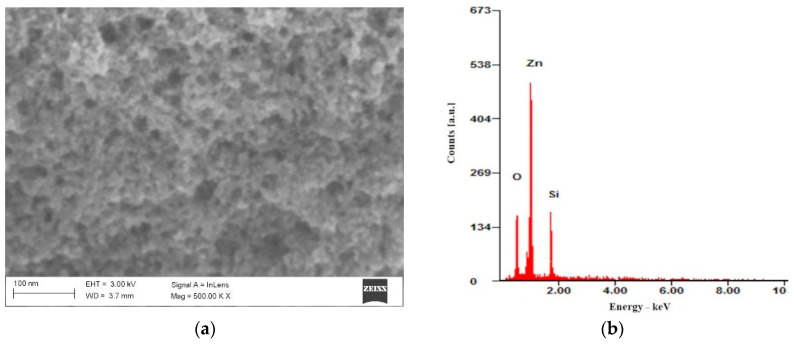
SEM image of ZnO nanoparticles (**a**) and it’s EDS spectrum (**b**).

The surface morphology of ZnO nanoparticles deposited on a silicon substrate was investigated using scanning electron microscopy (SEM), as illustrated in Figure 7a. The image reveals that the nanoparticle layer consists of aggregated structures of ZnO nanoparticles. Figure 7b displays the EDS spectrum of the ZnO nanoparticle layer, where peaks corresponding to zinc and oxygen are identified, along with a peak attributed to silicon from the substrate.

The SEM characteristics of the synthesized ZnO nanoparticles in this study align well with findings reported in the literature. For instance, a study published in *Nanoscale Research Letters* described the sol–gel synthesis of ZnO nanoparticles. In this study, the authors synthesized ZnO nanoparticles using a sol–gel method and characterized them using various techniques, including scanning electron microscopy (SEM) and energy-dispersive X-ray spectroscopy (EDS) [34]. The SEM images revealed that the nanoparticles formed aggregated structures, and the EDS analysis confirmed the presence of zinc and oxygen. These findings are consistent with our observations, where the ZnO nanoparticle layer consists of aggregated structures, and the EDS spectrum identifies peaks corresponding to zinc and oxygen.

**Figure 8:**
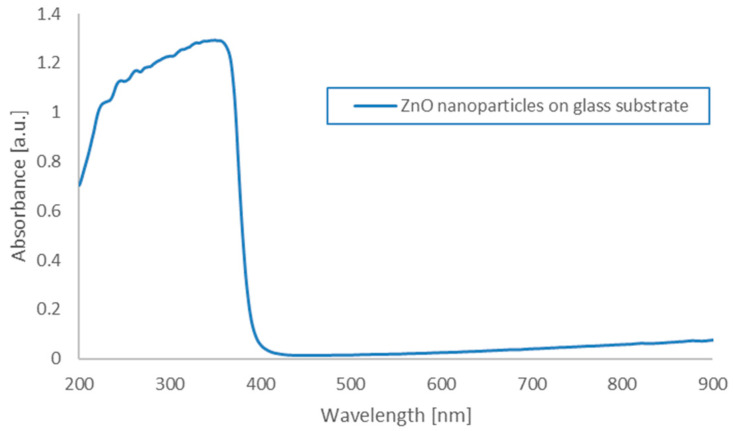
The absorbance spectrum as a function of wavelength for ZnO nanoparticles.

**Figure 9:**
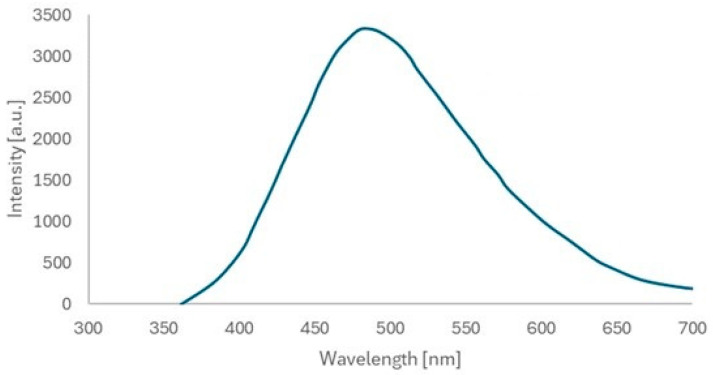
The PL spectrum as a function of wavelength for ZnO nanoparticles.

The absorbance of the nanoparticles was measured using a Thermo Scientific Evolution 220 spectrophotometer equipped with a xenon lamp with a wavelength range of 190 to 1110 nm. The absorbance of the ZnO nanoparticles deposited on the glass substrate was measured. The maximum was recorded at wavelengths of around 350 nm (characteristic for ZnO) (Figure 8). The photoluminescence (PL) spectrum of the as-prepared ZnO quantum dots in solid powder form was recorded, with the results presented in Figure 9. Typically, ZnO nanomaterials exhibit ultraviolet (UV) and visible emissions in their PL spectra. However, only visible emissions were observed in the case of the ZnO QDs synthesized in this study. Radiative transitions occur within the ZnO QDs upon UV light excitation, generally involving both UV and visible emissions, which compete. Beyond the excitation intensity, the UV-to-visible emission intensity ratio is primarily determined by the crystalline quality of the ZnO material. In general, larger ZnO nanostructures with near-perfect crystallinity tend to exhibit stronger UV emissions, whereas smaller structures with a high density of defects predominantly show stronger visible emissions.

**Figure 10:**
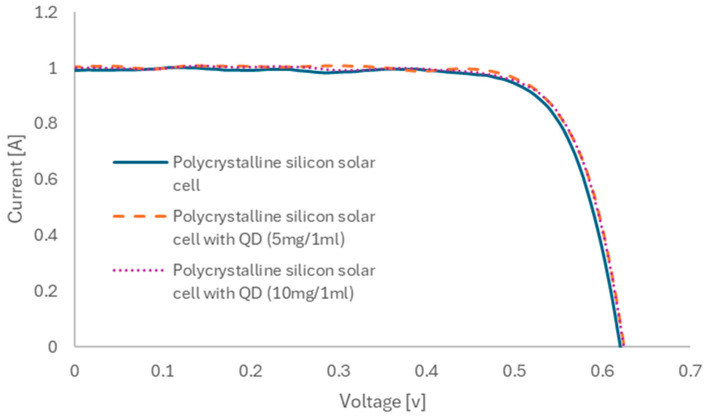
Current–voltage characteristics of the polycrystalline silicon solar cells with ZnO nanoparticles.

The current–voltage (I-V) characteristics of silicon solar cells, both with and without ZnO nanoparticles, were measured (Figure 10). Based on these measurements, the fundamental electrical parameters of the tested solar cells were calculated (Table 1). The solar cell with the highest efficiency in converting solar radiation into electricity was achieved with nanoparticles deposited from a 5 mg/mL solution. This solar cell exhibited an efficiency of 19.05%, with a short-circuit current of 1006 mA. The electrical performance of solar cells with nanoparticles deposited from a 10 mg/mL solution decreased slightly but remained superior to those without nanoparticles. In this case, the solar cell’s efficiency was 18.96%, with a short-circuit current of 1001 mA. In contrast, the efficiency of the solar cell without nanoparticles was 18.67%, with a short-circuit current of 988 mA. Compared to, e.g., the results obtained in the literature, when using silicon quantum dots achieved a current increase of about 1%, the use of ZnO quantum dots enabled an increase in the current value by about 1.8% [31]. According to the literature data, the use of a mixture of ZnO quantum dots with PMMA enabled the efficiency of silicon solar cells to increase from 14% to about 14.6% [35]. In this case, an increase from 18.67 to 19.05% was achieved. Further research will focus on the optimization of quantum dot deposition. It should be emphasized, however, that the authors started with a much higher efficiency of the base solar cells.

**Figure 11:**
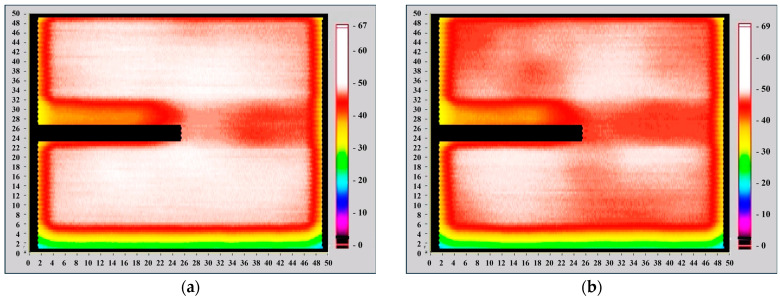
The distribution of the short circuit current over the silicon solar cell: (**a**) without ZnO nanoparticles, (**b**) with ZnO nanoparticles (5 mL/1 mg).

The Light Beam Induced Current (LBIC) scan method involves scanning a focused light beam across a solar cell while recording the corresponding short-circuit current at each position. Typically, LBIC systems utilize a highly focused beam, often as small as 0.1 mm in diameter, to achieve high spatial resolution. In contrast, the Corescan LBIC system operates at a lower spatial resolution due to its fixed beam diameter of 10 mm. When using light generated by a halogen lamp, the longer wavelengths allow for a deeper penetration into the cell, meaning the Corescan LBIC method primarily reflects variations in the bulk carrier lifetime across the material. Figure 11 shows that the surface of the silicon solar cell without ZnO quantum dots exhibited regions with a short-circuit current density ranging from 40 to 67 mA/cm^2^. After depositing ZnO quantum dots from a 5 mg/mL solution, the short-circuit current density increased from 40 to 69 mA/cm^2^. This suggests a potential positive effect of ZnO quantum dots on the conversion efficiency of silicon solar cells.

## 4. Conclusions

This study investigates the potential of zinc oxide (ZnO) quantum dots (QDs) to improve the performance of silicon-based solar cells. Despite its widespread use, silicon faces intrinsic limitations in capturing the entire solar spectrum, particularly in the ultraviolet (UV) range. As a result, a portion of the incoming solar energy is lost as heat rather than being converted into electricity. ZnO QDs, with their ability to absorb UV radiation and re-emit it in the visible spectrum, present a promising solution. ZnO QDs were synthesized using a cost-effective sol–gel method, which operates at low temperatures and requires minimal equipment. The structural analysis, conducted using transmission electron microscopy (TEM), revealed the polycrystalline nature of the ZnO nanoparticles, with diameters of less than 10 nm. Based on that, we can conclude that the chemical sol–gel method provides excellent control over the synthesis of ZnO QDs, ensuring reproducibility and a narrow size distribution, as confirmed by TEM imaging and the corresponding size histogram. These findings demonstrate the potential of this approach for producing uniform and scalable ZnO nanostructures, suitable for a wide range of applications in nanotechnology. Photoluminescence studies showed that the ZnO QDs exhibit visible emissions, enhancing their suitability for photovoltaic applications. ZnO QDs improved the short-circuit current and overall energy conversion efficiency when applied to polycrystalline silicon solar cells. The best-performing cell, with nanoparticles deposited from a 5 mg/mL solution, achieved an efficiency of 19.05%, compared to 18.67% for a cell without nanoparticles. These results indicate that incorporating ZnO QDs into solar cells can optimize their performance by capturing more solar spectrum.

Quantum dots (QDs) used to boost power conversion efficiency (PCE) in silicon-based solar cells include a variety of materials such as Si-QDs, CdS, ZnS, and ZnO QDs. Deposition methods—like spin coating and PECVD—are tailored to the host substrate. Studies show that PA Si-QDs improved short-circuit current (I_sc_) by up to 1.06%, while ZnO QDs enhanced it by around 1.8%. In the article, the authors achieved notable efficiency improvements in the silicon solar cells using ZnO quantum dots. Their application of ZnO QDs raised the cell efficiency from an initial 18.67% to 19.05%, highlighting the effectiveness of their specific approach. Comparatively, the previous methods using a ZnO and PMMA blend enhanced efficiency from 14.00% to 14.65%. These results underscore the authors’ success in optimizing quantum dot deposition techniques, demonstrating a significant efficiency gain in high-performance silicon solar cells.

The study demonstrated that ZnO QDs positively impact the electrical parameters of solar cells, including an increase in short-circuit current and overall energy conversion efficiency. Although the results show promising improvements, it should be noted that the efficiency increase is not drastic, and the performance remains lower compared to other materials, such as Si-QDs or a ZnO QDs/PMMA blend. Additionally, the results suggest that further optimization of the quantum dot deposition process is needed to achieve even better efficiency and stability in solar cells. Future research should focus on further optimizing ZnO QD deposition techniques, such as controlling their size and shape, as well as improving their deposition quality on the substrate. Increasing spatial resolution and improving the absorption coefficient over a broader spectral range could also contribute to further improvements in solar cell efficiency. Long-term studies on the durability of ZnO QD-based solar cells should also be conducted to evaluate their stability under operational conditions.

The study highlights the feasibility of integrating ZnO QDs into existing photovoltaic manufacturing processes and opens avenues for further research into optimizing quantum dot properties for solar energy applications. This work significantly contributes to the field of photovoltaics by applying zinc oxide in the form of quantum dots (ZnO QDs), which show considerable improvements in the energy conversion efficiency of silicon-based solar cells. The use of the sol–gel method for synthesizing ZnO QDs provides an effective way to produce uniform and high-quality nanostructures, which could be widely applied in the industrial production of solar cells.

## Figures and Tables

**Table 1:** Summary of electrical properties of the polycrystalline silicon solar cells with ZnO nanoparticles.

Samples	V_OC_ [V]	I_SC_ [mA]	Efficiency [%]
Polycrystalline silicon solar cell	0.621	988	18.67
Polycrystalline silicon solar cell with QD (5 mg/1 mL)	0.624	1006	19.05
Polycrystalline silicon solar cell with QD (10 mg/1 mL)	0.623	1001	18.96

## Data Availability

The original contributions presented in the study are included in the article, and further inquiries can be directed to the corresponding authors.

## Abbreviations

The following abbreviations are used in this manuscript:

ZnO	Zinc Oxide
QDs	Quantum Dots
UV	Ultraviolet
HRTEM	High-Resolution Transmission Electron Microscopy
SEM	Scanning Electron Microscopy
TEM	Transmission Electron Microscopy
EDS	Energy Dispersive Spectroscopy
I-V	Current–Voltage
PCE	Power Conversion Efficiency
LBIC	Light Beam Induced Current
I_SC_	Short-Circuit Current
V_OC_	Open-Circuit Voltage
